# *Dendrobium officinale* Polysaccharide Promotes Infected Wound Healing Through SIRT1-Regulated HMGB1-K29 Deacetylation and NF-κB Inhibition

**DOI:** 10.5812/ijpr-169909

**Published:** 2026-05-26

**Authors:** Yutong Wu, Mohd Hasmizam Razali, Wentao Wu

**Affiliations:** 1 Department of Otolaryngology Head and Neck Plastic Surgery and Repair, Honghui Hospital, Xi'an Jiaotong University, Xi'an, China; 2 Advanced Nanomaterials Research Group, Faculty of Science and Marine Environment, Universiti Malaysia Terengganu, 21030 Kuala Nerus, Terengganu, Malaysia; 3 Faculty of Science and Marine Environment, Universiti Malaysia Terengganu, 21030 Kuala Nerus, Terengganu, Malaysia

**Keywords:** Dendrobium Officinale Polysaccharide, SIRT1, HMGB1, NF-κB Signaling Pathway, Wound Healing

## Abstract

**Background:**

Two major challenges frequently encountered during wound healing are excessive inflammatory responses and bacterial infection. *Dendrobium officinale* polysaccharide (DOP), a major bioactive constituent of *Dendrobium officinale*, has shown potential for promoting infected wound healing and tissue repair.

**Objectives:**

This study evaluated the therapeutic efficacy of DOP in an infected murine wound model, with a specific focus on the role of the SIRT1/HMGB1/NF-κB signaling axis.

**Methods:**

Mouse full-thickness skin defect models were established, and animals were divided into four cohorts: control, infected model, DOP-treated, and DOP plus the SIRT1-specific inhibitor EX527. All groups except the control group were inoculated with *Staphylococcus aureus*. The primary endpoint was wound closure, quantified as the remaining wound surface area on day 14. Secondary outcomes included body weight changes, histopathological injury scores, inflammatory cytokine protein secretion and mRNA transcript levels, SIRT1 expression, HMGB1 subcellular localization, IκBα degradation, and NF-κB p65 phosphorylation.

**Results:**

For the primary endpoint, animals receiving DOP had markedly smaller unclosed wound areas on postoperative day 14 than untreated infected animals. Secondary analyses showed that DOP improved body weight recovery and reduced inflammatory cytokine secretion and corresponding mRNA transcript levels. Mechanistically, DOP increased SIRT1 protein levels, promoted HMGB1 deacetylation at the K29 residue with consequent nuclear sequestration, and suppressed IκBα proteolysis and NF-κB p65 phosphorylation. These protective effects were reversed by the SIRT1 inhibitor EX527.

**Conclusions:**

By activating SIRT1, DOP promotes K29 deacetylation of HMGB1 and retains this alarmin in the nucleus. The resulting suppression of NF-κB reduces inflammatory injury and counteracts the adverse effects of bacterial infection on wound repair. These findings provide an experimental basis for the potential application of DOP in the management of infected wounds.

## 1. Background

Compromised wound repair is a pervasive clinical concern, and its pathophysiological basis is fundamentally intertwined with unresolved inflammatory activity (1). Although this association is widely acknowledged, the precise molecular mechanisms remain incompletely elucidated (2). Therefore, in-depth exploration of the regulatory mechanisms governing wound healing has substantial research value.

In recent years, natural plant-derived bioactive substances have attracted considerable interest in wound repair because of their multitarget regulatory effects, high biosafety, and low risk of inducing drug resistance. For example, green-synthesized copper nanoparticles prepared with *Lupinus arcticus* extract have been shown to exert antibacterial, anti-inflammatory, and proangiogenic activities and to effectively promote wound healing, providing experimental evidence and a research reference for applying natural product-derived bioactive components to refractory wounds (3). As a key bioactive constituent of the traditional Chinese medicine *Dendrobium officinale*, DOP exhibits potent anti-inflammatory and pro-healing properties (4). These characteristics make it an ideal candidate for investigating wound healing mechanisms. However, whether DOP exerts its therapeutic effects by regulating key inflammatory signaling pathways remains unclear.

In inflammatory regulation, HMGB1 functions as an important driver of the late-phase inflammatory response (5). The functional activity of HMGB1 is tightly regulated by acetylation (6). Under stressful conditions, such as infection, HMGB1 undergoes acetylation at specific lysine residues, including K29, which induces nuclear export and subsequent extracellular secretion (7-9).

Sirtuin 1 (SIRT1), an NAD^+^-dependent deacylase, occupies a central regulatory position in inflammatory responses (10). Experimental evidence indicates that SIRT1 directly catalyzes HMGB1 deacetylation, thereby retaining it within the nucleus and preventing its cytosolic migration and subsequent downstream inflammatory amplification.

Building on this foundation, we investigated the potential association between DOP and this regulatory axis. Previous studies have shown that DOP confers anti-inflammatory and antioxidant benefits in experimental settings, including diabetic wound models and renal fibrosis (11, 12). In particular, in a diabetic nephropathy model, DOP exerted antifibrotic effects by regulating the SIRT1 signaling pathway (12).

## 2. Objectives

Based on this background, the present study used a *Staphylococcus aureus*-infected mouse wound model to systematically evaluate the therapeutic efficacy of DOP and to investigate its regulatory effects on the SIRT1/HMGB1/NF-κB signaling axis.

## 3. Methods

### 3.1. Animals

A total of 12 healthy 8-week-old male BALB/c mice (body weight, 22 - 24 g) were purchased from SPF Biotechnology (Beijing). Animals were maintained under controlled conditions of 22 ± 2°C, 50 ± 10% humidity, and a 12-hour light/dark cycle (13, 14). They were provided ad libitum access to food and water. After 7 days of acclimatization, mice with consistent body weights were numbered and randomly assigned to 4 experimental groups using a random number generator by an investigator not involved in animal care (W.T.W.). Allocation was performed by another researcher (Y.T.W.) using sequentially numbered opaque envelopes to conceal the sequence until assignment. The outcome assessor was blinded to group allocation during the experiment and data analysis. Each group contained 3 mice.

### 3.2. Establishment and Grouping of *Staphylococcus Aureus*-Infected Wounds in Mice

#### 3.2.1. Culture and Preparation of *Staphylococcus Aureus* Suspension

Cryopreserved Staphylococcus aureus (ATCC 6583) was revived by inoculation onto TSA solid medium and incubated at 37°C for 1 day until distinct individual colonies were visible. A single morphologically typical colony was selected and transferred into a liquid medium for subculture. The culture was incubated in a shaking incubator at 37°C with agitation at 200 rpm for 12 hours (15). After serial dilution, the viable bacterial concentration was determined using the plate counting method. The bacterial suspension was finally adjusted to 4 × 10^6^ CFU/mL for subsequent use.

#### 3.2.2. Animal Grouping and Treatment

Surgical anesthesia was induced by exposure to 5% isoflurane vapor in a sealed induction chamber and then maintained by continuous delivery of 2% isoflurane throughout the procedure (16). After disinfection of the dorsal skin, full-thickness excisional wounds were created on the backs of the mice using a 6-mm skin biopsy punch (17). Mice were kept on a 37°C heating pad until full recovery from anesthesia. All wounds were kept uncovered, and each mouse was housed individually to avoid secondary wound damage. The general condition of each mouse was monitored daily, including signs of lethargy, reduced activity, and excessive weight loss (> 20%). Predefined humane endpoints, including severe local infection, impaired mobility, and moribund status, were established in advance. No additional analgesics were administered after surgery because this superficial infected wound model was expected to cause only mild procedural pain and complied with institutional animal care guidelines. No adverse events, self-mutilation, severe wound deterioration, animal death, or animal exclusion occurred throughout the experiment.

After successful model establishment, the 12 animals were distributed across 4 experimental cohorts (3 animals per cohort) by computer-assisted randomization: sham control group (Sham), infection model group (Infection), *Dendrobium officinale* polysaccharide treatment group (DOP; Shaanxi Sinot Biotechnology Co., Ltd.; No. 2115-91-5), and DOP combined with EX527 group (DOP + EX527; EX527 is a specific SIRT1 inhibitor; Beyotime, SC0281 - 5mg). Except for the sham control group, all animals received topical application of an 80 μL bacterial suspension (4 × 10^6^ CFU/mL) onto the wound surface to establish an infected wound model (18). The sham control group received 80 μL phosphate-buffered saline (PBS) instead.

Both DOP and EX527 were dissolved in normal saline. Based on previous literature, the administration doses were set at 400 mg/kg for DOP and 2 mg/kg for EX527 (19). Drug interventions were administered once daily for 14 days, starting immediately after infection/wounding on day 0. Mice in the DOP group received DOP by intragastric administration, whereas mice in the DOP + EX527 group received the same dose of DOP intragastrically along with an intraperitoneal injection of EX527. To exclude interference from procedural factors, the sham control and infection model groups received equal volumes of normal saline by intragastric administration and intraperitoneal injection. The DOP group also received an additional intraperitoneal injection of an equivalent volume of normal saline. The administration volume for all intragastric procedures was 10 mL/kg. The treatment period lasted 2 weeks. The primary outcome was residual wound area on day 14. On days 0, 3, 7, and 14 of the experiment, body weight changes and wound healing progression were recorded for each group.

### 3.3. Histopathological Analysis of Wound Tissues with H&E Staining

Twenty-four hours after treatment, animals were euthanized by cervical dislocation. Full-thickness wound specimens were harvested, fixed in 4% paraformaldehyde, and embedded in paraffin. Subsequently, 4-μm sections were cut, deparaffinized, rehydrated, and stained with H&E (Servicebio, G1076). Histopathological assessment of inflammatory cell infiltration, tissue necrosis, and granulation tissue formation was independently conducted by 2 professional pathologists who were masked to treatment assignment to minimize observer bias (20).

### 3.4. Enzyme-Linked Immunosorbent Assay

Wound tissue fragments were washed with chilled phosphate buffer (0.01 M, pH 7.0 - 7.2), blotted, weighed, finely minced, and mechanically disrupted in a 9-fold volume of the same buffer using a glass-walled homogenizer in an ice bath (21). The resulting homogenates were centrifuged, and the supernatants were analyzed for IL-6, IL-1β, and TNF-α levels using specific ELISA kits (JONLNBIO, JL20268, JL18442, and JL10484) according to the manufacturer's instructions.

### 3.5. Western Blot

Whole-cell protein was extracted from tissue specimens in RIPA buffer (Solarbio, R0010) supplemented with protease and phosphatase inhibitor cocktails (Beyotime, P1045). Lysates were kept on ice for 15 minutes before being divided into 2 equal portions. The first portion was clarified at 14,000 × g for 15 minutes at 4°C to yield the total tissue extract. The second portion underwent nuclear-cytoplasmic fractionation using a commercial separation kit (Thermo Fisher, 78835) according to the enclosed protocol.

Total protein content across all specimens was quantified using a BCA-based colorimetric kit (Solarbio, PC0020). Each sample was combined with SDS loading buffer and heat-denatured at 95°C for 5 minutes before separation on 10% polyacrylamide gels under denaturing conditions (22). The resolved proteins were subsequently transferred onto PVDF membranes (22). After blocking with 5% skim milk for 2 hours at room temperature, membranes were probed overnight at 4°C with the following primary antibodies (22, 23): SIRT1 (Proteintech, RMX00009, rabbit monoclonal, 1:1000), IκBα (Proteintech, 10268 - 1-AP, rabbit polyclonal, 1:1000), NF-κB p65 (Proteintech, 10745 - 1-AP, rabbit polyclonal, 1:1000), HMGB1 (Proteintech, 10829 - 1-AP, rabbit polyclonal, 1:1000), β-tubulin (Proteintech, 10094 - 1-AP, rabbit polyclonal, 1:1000), and Histone H3 (Proteintech, 17168 - 1-AP, rabbit polyclonal, 1:1000). Whole-tissue lysates were used to detect total protein levels of SIRT1, IκBα, NF-κB p65, and HMGB1, whereas fractionated samples were used to compare HMGB1 expression in the nuclear and cytoplasmic compartments.

After washing, a horseradish peroxidase-labeled goat anti-rabbit secondary antibody (Servicebio, GB23303; 1:5000, 60 minutes, ambient temperature) was applied. Immunoreactive bands were detected by enhanced chemiluminescence on a BIO-RAD Universal Hood II system, and band densities were quantified using ImageJ (5).

### 3.6. Immunofluorescence Staining

After deparaffinization and rehydration, tissue sections underwent membrane permeabilization in 1% Triton X-100/PBS for 10 minutes, followed by antigen unmasking and blockade of nonspecific binding with 3% bovine serum albumin at room temperature for 30 minutes. The sections were then incubated overnight at 4°C with a primary antibody targeting acetylated HMGB1 at lysine 29 (acetyl-HMGB1-K29; Abclonal Technology, A22709, rabbit polyclonal, 1:1000) (24). After removal of the primary antibody, the sections were washed 3 times with PBS and then incubated with FITC-conjugated goat anti-rabbit IgG (Servicebio, GB22303, 1:200) for 30 minutes at room temperature (25). Nuclei were counterstained with DAPI (Servicebio, G1012, 1:1000) for 5 minutes at room temperature, and coverslips were applied before image acquisition. Image screening and quantitative analysis were completed by researchers blinded to group allocation using ImageJ software (26).

### 3.7. RNA Extraction and Quantitative Real-time PCR

Tissue RNA was isolated using a commercial extraction reagent (Solarbio, R1200) according to the supplier's guidelines. Optical absorbance at 260 and 280 nm, measured using a NanoDrop 2000 instrument (Thermo Scientific), was used to evaluate RNA yield and integrity. One microgram of RNA per sample was converted to first-strand cDNA using a dedicated reverse-transcription kit (Yeasen Biotechnology, 11119ES60) (27). Quantitative real-time PCR was performed on an ABI 7300 system with SYBR Green master mix (Yeasen Biotechnology, 11203ES03). All reactions were performed in triplicate for both target genes and the housekeeping gene GAPDH. Transcript abundance was normalized to GAPDH and expressed as fold change relative to the sham group using the comparative ΔΔCt calculation. Table S1 in Supplementary File presents the primer details (28).

### 3.8. Statistical Analysis

All statistical computations were performed using GraphPad Prism version 10.1. Continuous outcomes are reported as mean ± SD. Before group comparisons, distributional assumptions were verified using the Shapiro-Wilk normality test and the Levene homogeneity-of-variance test. Between-group differences were evaluated by one-way ANOVA, and pairwise comparisons were adjusted using the Tukey post hoc procedure. A P value < 0.05 was considered statistically significant. For repeated measures, including body weight and wound area, data are presented as descriptive statistics.

## 4. Results

### 4.1. DOP Promotes Body Weight Recovery and Wound Healing in Mice With Infected Wounds

**Figure 1.**
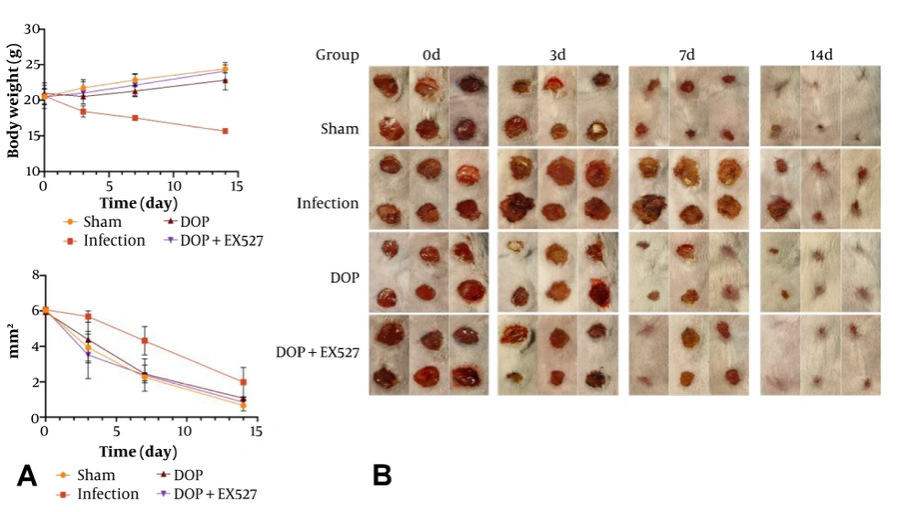
DOP ameliorates body weight recovery and promotes wound healing in infected mice. Body weight changes in mice from the Sham, Infection, DOP, and DOP + EX527 groups were recorded at baseline and on days 0, 3, 7, and 14 to evaluate the effect of DOP on weight recovery in infected mice. A, Quantitative analysis of wound healing in each group from day 0 to day 14 (unit: mm^2^); B, Representative wound images of mice from the Sham, Infection, DOP, and DOP + EX527 groups were captured at the same intervals to visualize wound closure dynamics. Values are expressed as mean ± SD (n = 3/group). Descriptive statistics only; no inferential testing was performed.

To characterize the effects of DOP on recovery in bacterially infected mice, body mass was monitored from baseline through days 3, 7, and 14. Progressive weight gain was observed in all cohorts except the Infection group. Animals co-administered DOP and EX527 exhibited a slower rate of weight recovery than those treated with DOP alone (Figure 1A). Wound closure progressed in all groups throughout the observation period. For the primary endpoint, the unclosed wound area on day 14 was substantially smaller in DOP-treated mice than in the Infection cohort, whereas the DOP + EX527 group showed moderately attenuated healing compared with the DOP-only group (Figure 1B). Quantitative analysis of wound-healing images is presented in Figure 1.

### 4.2. DOP Alleviates Histopathological Damage in Infected Wounds

**Figure 2.**
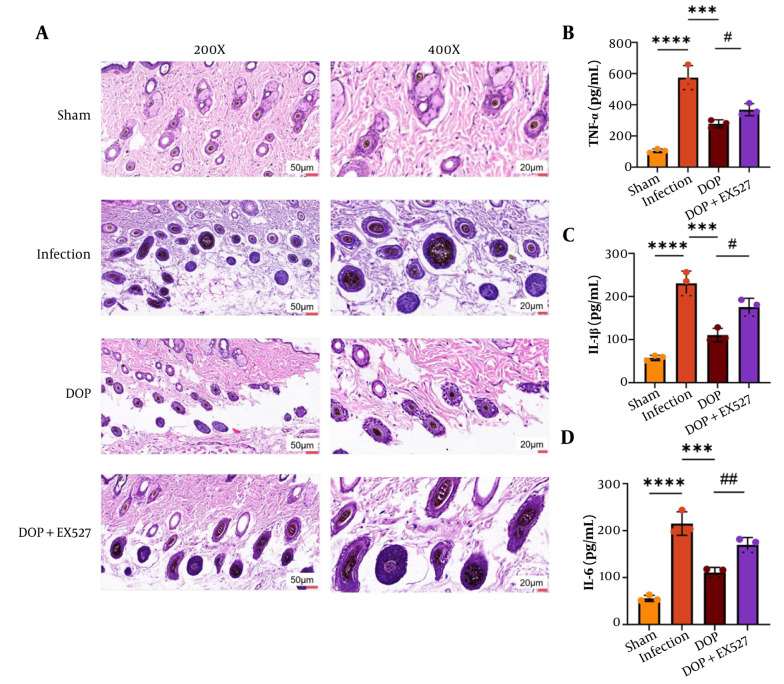
DOP modulates histopathological damage and inflammatory cytokine expression in infected wounds. (A) H&E-stained sections of wound tissues obtained from the Sham, Infection, DOP, and DOP + EX527 groups showing inflammatory cell infiltration, tissue necrosis, and granulation tissue formation. Images were captured at 200 × (50 μm scale bar) and 400 × (20 μm scale bar). (B-D) Levels of TNF-α (B), IL-1β (C), and IL-6 (D) in wound tissue homogenates were quantified by ELISA. Values are expressed as mean ± SD (n = 3/group). Group comparisons were made using 1-way ANOVA. ***P < 0.001, ****P < 0.0001 vs Infection group; #P < 0.05, ##P < 0.01 vs DOP group.

To assess the influence of DOP on wound tissue pathology at the histological level, H&E-stained sections were examined (Figure 2A). Pathological analysis showed that the sham group had no significant inflammatory cell infiltration or tissue necrosis and exhibited well-developed granulation tissue. In contrast, the Infection group showed marked inflammatory infiltration, obvious tissue necrosis, and sparse granulation tissue. Compared with the Infection group, DOP treatment significantly reduced inflammatory cell infiltration and tissue necrosis while increasing granulation tissue. Conversely, the addition of EX527 reversed these effects, resulting in increased inflammation, aggravated necrosis, and reduced granulation tissue compared with the DOP group.

### 4.3. DOP Reduces Pro-Inflammatory Cytokine Levels in Infected Wounds

We further examined the regulatory role of DOP in modulating local cytokine production in infected wound tissues, which closely correlated with the histopathological changes described above. Compared with the sham group, infected animals showed markedly increased release of the pro-inflammatory mediators TNF-α, IL-1β, and IL-6. DOP intervention significantly reduced the overproduction of these inflammatory factors, whereas combined treatment with DOP and the SIRT1 inhibitor EX527 markedly reversed the anti-inflammatory activity of DOP (Figure 2B-D). Similar trends were observed for all 3 cytokines examined, indicating that DOP alleviates local inflammation by suppressing these mediators, an effect that EX527 appeared to antagonize.

### 4.4. DOP Suppresses the NF-κB Pathway by Activating SIRT1-Mediated HMGB1 Deacetylation and Nuclear Retention

**Figure 3.**
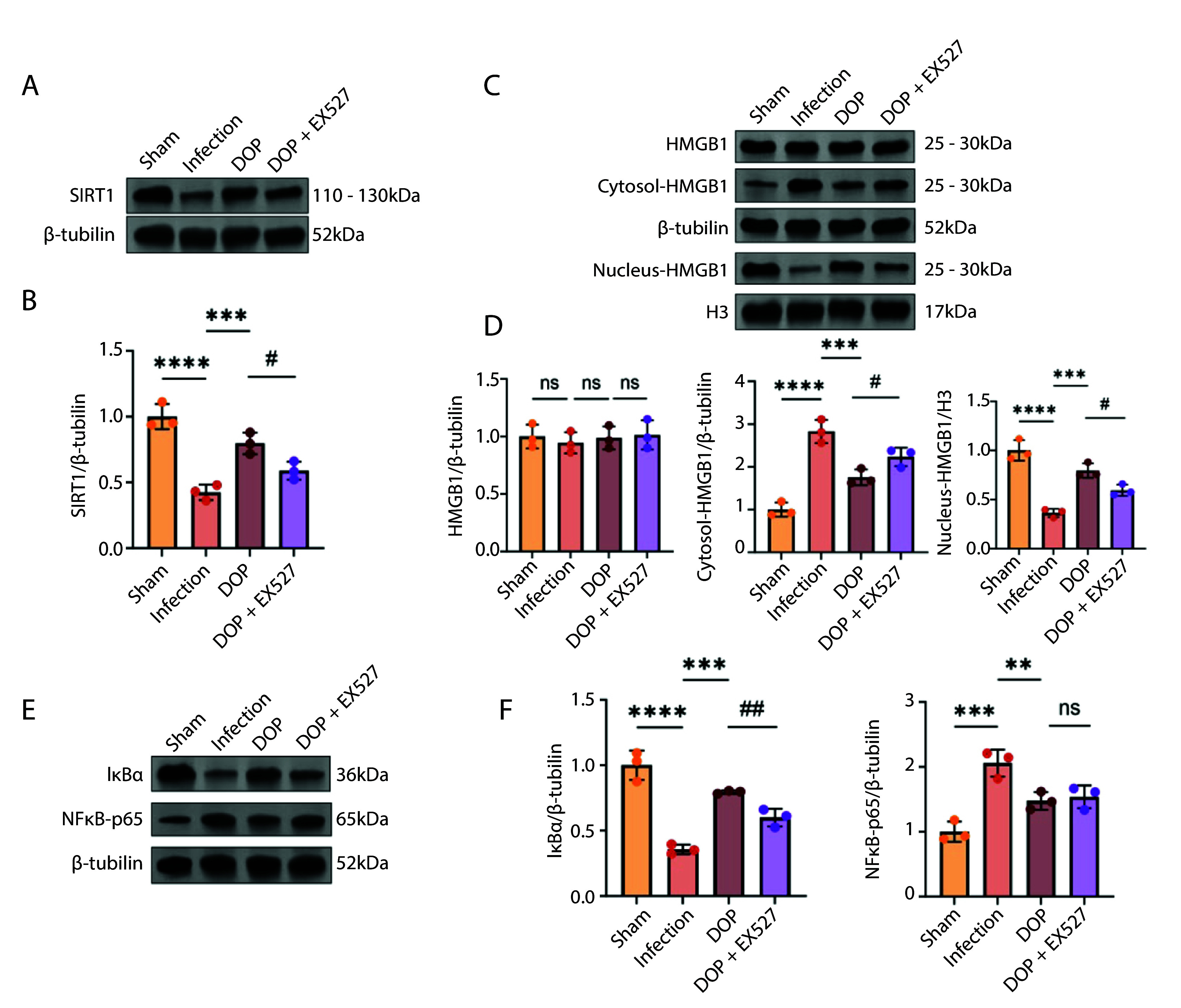
DOP activates SIRT1 to mediate HMGB1 deacetylation/nuclear retention and suppresses the NF-κB pathway. (A-B) SIRT1 protein levels detected by immunoblotting (A) and quantified (B), demonstrating that DOP upregulates SIRT1 expression, an effect reversed by the SIRT1 inhibitor EX527. (C-D) Immunoblot assessment of total HMGB1, cytosolic HMGB1, and nuclear HMGB1 (C), with densitometry (D), indicating that DOP promotes HMGB1 deacetylation and nuclear retention via SIRT1, thereby inhibiting its cytosolic translocation. (E-F) Immunoblot detection of IκBα and phosphorylated NF-κB p65 (E) and corresponding quantification (F). Data are presented as mean ± SD (n = 3/group). Analysis used 1-way ANOVA with Tukey post hoc test. ** P < 0.01, *** P < 0.001, **** P < 0.0001 vs indicated groups; # P < 0.05, ## P < 0.01 vs DOP group; ns indicates not significant.

To investigate the mechanism by which DOP modulates inflammation in infected wounds, we examined the SIRT1/HMGB1/NF-κB pathway. We first assessed SIRT1 expression. Western blotting (Figure 3A) and quantitative analysis (Figure 3B) indicated that SIRT1 protein levels were markedly decreased in the Infection group. DOP treatment markedly increased SIRT1 expression, whereas the DOP + EX527 group significantly reversed this activating effect of DOP (P < 0.001 and P < 0.05). Because HMGB1 acetylation is associated with its cellular localization, we further examined HMGB1 acetylation and subcellular localization. Western blotting detected total HMGB1, cytosolic HMGB1, and nuclear HMGB1 (Figure 3C). Quantitative analysis revealed increased cytosolic HMGB1 and reduced nuclear HMGB1 in the Infection group (Figure 3D). DOP treatment reduced cytosolic HMGB1 and increased nuclear HMGB1, whereas DOP + EX527 reversed these changes (ns indicates not significant; P < 0.05), suggesting that DOP promotes SIRT1-mediated HMGB1 deacetylation and nuclear retention. Given the reported link between the NF-κB pathway and HMGB1 deacetylation, we next evaluated NF-κB signaling. Western blotting (Figure 3E) detected IκBα and NF-κB p65 phosphorylation levels. Quantitative analysis (Figure 3F) showed increased IκBα degradation and significantly elevated NF-κB p65 phosphorylation in the Infection group (P < 0.001). DOP treatment attenuated both IκBα degradation and NF-κB p65 phosphorylation, whereas DOP + EX527 antagonized this inhibitory effect of DOP. Collectively, these results demonstrate that DOP activates SIRT1 to mediate HMGB1 deacetylation and nuclear retention, thereby inhibiting the NF-κB pathway and modulating inflammation in infected wounds.

### 4.5. DOP Potentially Exerts Its Anti-Inflammatory Effects Through SIRT1-Mediated Deacetylation of HMGB1 at K29

**Figure 4.**
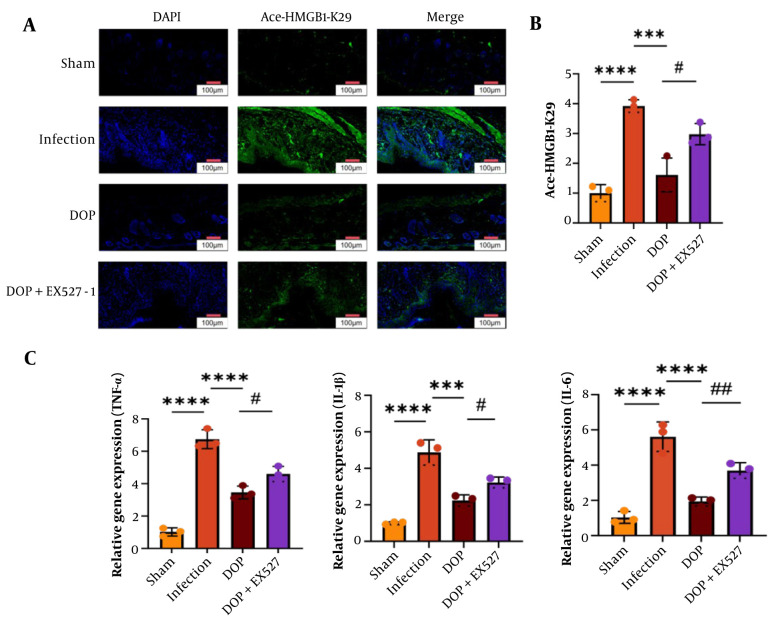
DOP exerts anti-inflammatory effects via SIRT1-mediated HMGB1-K29 deacetylation. (A) Immunofluorescence staining of acetylated HMGB1-K29 (Ace-HMGB1-K29) in wound tissues from the Sham, Infection, DOP, and DOP + EX527 groups. (B) Quantitative analysis of Ace-HMGB1-K29 levels. (C) mRNA levels of TNF-α, IL-1β, and IL-6 quantified by real-time PCR. Data are presented as mean ± SD (n = 3/group). Statistical analysis was performed using 1-way ANOVA. *** P < 0.001, **** P < 0.0001 vs indicated groups; # P < 0.05, ## P < 0.01 vs DOP group.

To determine whether the anti-inflammatory action of DOP involves SIRT1-mediated deacetylation of HMGB1 at lysine 29 (K29), we examined the expression of acetylated HMGB1-K29 (Ace-HMGB1-K29) in infected wounds. Immunofluorescence staining (Figure 4A) showed that Ace-HMGB1-K29 fluorescence intensity was markedly elevated in the Infection group, substantially reduced by DOP treatment, and restored by co-treatment with EX527. Quantification of Ace-HMGB1-K29 levels (Figure 4B) confirmed these observations, showing a significant increase in the Infection group and marked downregulation after DOP treatment, which was reversed by EX527. Subsequently, real-time PCR was used to measure mRNA levels of TNF-α, IL-1β, and IL-6 (Figure 4C). These analyses demonstrated significant upregulation in the Infection group.

## 5. Discussion

Defective wound repair driven by persistent inflammation represents a clinically challenging problem that urgently requires effective solutions. Beyond delaying tissue regeneration, uncontrolled inflammatory activity reduces patient quality of life and imposes a growing burden on medical systems. The current first-line approach for *S. aureus*-infected wounds, combining systemic antibiotics with surgical tissue removal, has inherent drawbacks. Debridement itself causes secondary tissue injury and procedural discomfort, and the worsening global problem of antimicrobial resistance steadily erodes the reliability of antibiotic regimens. Given these constraints, the research community has increasingly focused on deciphering the molecular basis of wound healing and identifying new targets for anti-inflammatory intervention.

In this context, traditional Chinese medicine-derived therapies have shown considerable efficacy in wound repair, including attenuation of inflammatory cascades and reduction of oxidative burden, as evidenced by studies of various herbal extracts and bioactive compounds (29, 30). Among them, DOP, the primary bioactive component of *Dendrobium officinale*, has received increasing attention. Previous work indicates that DOP has significant anti-inflammatory activity and tissue repair capabilities (31). Nevertheless, whether DOP exerts its effects by regulating key inflammatory signaling pathways and what the precise mechanism of action is remain unclear. To address this question, we developed a mouse model of *S. aureus*-infected wounds, systematically evaluated the therapeutic efficacy of DOP, and investigated its regulatory effects on the SIRT1/HMGB1/NF-κB axis.

The experimental findings demonstrated that this animal model successfully recapitulated the pathological characteristics of clinical infected wounds. DOP treatment significantly accelerated wound closure, improved the body weight trajectory of infected mice, and improved local tissue pathology, as indicated by reduced inflammatory cell infiltration, diminished tissue necrosis, and enhanced granulation tissue formation. Notably, the protective effects of DOP were significantly attenuated after treatment with EX527, a specific inhibitor of SIRT1, indicating that SIRT1 activation is a critical mediator of the therapeutic effects of DOP.

SIRT1, an NAD^+^-dependent deacetylase, occupies a central position in inflammation modulation (10, 32, 33). Accumulating evidence has confirmed that SIRT1 directly mediates HMGB1 deacetylation, promoting its nuclear retention and thereby inhibiting its cytoplasmic translocation and subsequent inflammatory responses (34, 35). As a key late-stage inflammatory mediator, HMGB1 function is precisely regulated by acetylation modification (36, 37). Once specific lysine positions, including K29 within HMGB1, become hyperacetylated, the protein undergoes nuclear egress, accumulates in the cytosol, and is ultimately secreted into the extracellular space (37, 38). Once released into the extracellular milieu, HMGB1 engages TLR receptors to trigger NF-κB activation, driving a robust surge in downstream inflammatory cytokines, including TNF-α, IL-1β, and IL-6 (39).

At the molecular level, our Western blot analysis yielded important findings. DOP intervention significantly increased SIRT1 protein expression while reducing HMGB1 acetylation at the K29 residue. In particular, DOP treatment effectively inhibited IκBα degradation and markedly decreased NF-κB p65 phosphorylation. These results suggest that DOP may regulate HMGB1 deacetylation by activating SIRT1, ultimately suppressing excessive activation of the NF-κB signaling pathway.

To validate this mechanism, we performed reverse-validation experiments. After treatment with EX527, the promoting effect of DOP on HMGB1 deacetylation and its inhibitory effect on the NF-κB signaling pathway were significantly reduced. This finding further confirms the central role of SIRT1 in the mechanism of action of DOP.

Combined immunofluorescence staining and ELISA assays showed that DOP markedly decreased acetylated HMGB1-K29 in infected wound tissues, an effect that was reversed by EX527. Meanwhile, qRT-PCR gene expression analysis demonstrated that DOP suppressed the transcription of TNF-α, IL-1β, and IL-6.

This study is exploratory mechanistic research. A small sample size (n = 3/group) was used in accordance with the 3R principles for animal welfare and because this study served as a proof-of-concept investigation to preliminarily delineate the core mechanism. All animals completed the study and were included in the final analysis. Nevertheless, the limited sample size without a priori power calculation may restrict the generalizability of the findings. In addition, bacterial burden in wound tissue was not quantified; therefore, no conclusion regarding direct antimicrobial effects can be drawn from the current data. In future studies, we will perform rigorous sample size calculations, increase the number of animals, and construct additional infection models to further validate our findings.

Taken together, this work provides a systematic mechanistic account of how DOP accelerates infected wound healing through coordinated regulation of the SIRT1/HMGB1/NF-κB cascade. The concordance between molecular readouts, including SIRT1 induction, HMGB1-K29 deacetylation, and NF-κB attenuation, and functional outcomes, including wound closure and cytokine suppression, remained consistent across all experimental arms and supports the proposed mechanism.

### 5.1. Conclusions

The findings of this investigation demonstrate that DOP significantly promotes the healing of *S. aureus*-infected wounds, as evidenced by a substantially reduced unclosed wound surface on day 14, recovery of body weight, and alleviation of tissue-level pathological injury caused by infection. At the molecular level, DOP upregulates SIRT1 expression, promotes HMGB1 deacetylation at the K29 site, and enhances its nuclear retention, thereby inhibiting its translocation to the cytoplasm and subsequent release. This action further blocks IκBα degradation and NF-κB p65 phosphorylation, attenuating overactivation of the NF-κB signaling pathway. Concurrently, DOP significantly reduces the transcription of TNF-α, IL-1β, and IL-6. Notably, these protective effects were all reversed by the SIRT1-specific inhibitor EX527.

This study systematically elucidates the molecular pathway through which DOP promotes the healing of infected wounds by regulating the SIRT1/HMGB1/NF-κB signaling axis. These insights advance knowledge of the anti-inflammatory actions of DOP and provide a basis for developing novel DOP-based wound treatment strategies.

## Data Availability

The data that support the findings of this study are available from the corresponding author upon reasonable request.
